# Factors During Pregnancy, Delivery and Birth Affecting Global Quality of Life of the Adult Child at Long-term Follow-up. Results from the Prospective Copenhagen Perinatal Birth Cohort 1959-61

**DOI:** 10.1100/tsw.2005.112

**Published:** 2005-12-01

**Authors:** Søren Ventegodt, Trine Flensborg-Madsen, Niels Jørgen Andersen, Joav Merrick

**Affiliations:** ^1^ Nordic School of Holistic Medicine, Teglgårdstræde 4-8, DK-1452 Copenhagen K, Denmark; ^2^ Quality of Life Research Center, Teglgårdstræde 4-8, DK-1452 Copenhagen K, Denmark; ^3^ Quality of Life Research Clinic, Teglgårdstræde 4-8, DK-1452 Copenhagen K, Denmark; ^4^ The Scandinavian Foundation for Holistic Medicine, Sandvika, Norway; ^5^ Norwegian School of Management, Sandvika, Norway; ^6^ National Institute of Child Health and Human Development, Faculty of Health Sciences, Ben Gurion University of the Negev, Beer-Sheva, Israel; ^7^ Center for Multidisciplinary Research in Aging, Faculty of Health Sciences, Ben Gurion University of the Negev, Beer-Sheva, Israel; ^8^ Division of Pediatrics, Faculty of Health Sciences, Ben Gurion University of the Negev, Beer-Sheva, Israel; ^9^ Office of the Medical Director, Division for Mental Retardation, Ministry of Social Affairs, Jerusalem, Israel

**Keywords:** Birth cohort, longitudinal study, maternal health, child health, development, global quality of life, QOL, holistic medicine, SEQOL, painkillers, psychopharmacology, Denmark

## Abstract

This paper presents a prospective cohort study, where we explore associations between pregnancy, delivery and the global quality of life (QOL) of the adult child 31-33 years later. The data is from the Copenhagen Perinatal Birth Cohort 1959-61 using two sets of questionnaires send to 7,222 persons: one filled out by physicians during pregnancy and delivery, while the follow-up questionnaire was completed by the adult children 31-33 years later. The main outcome measures were objective factors describing pregnancy and delivery along with global quality of life, including: Well-being, life satisfaction, happiness, fulfilment of needs, experience of life's temporal and spatial domains, expression of life's potentials and objective measures. Results showed two main factors in pregnancy that seemed to be associated with a reduced quality of life for the child 31-33 years later: the mother's smoking habits and the mother's medication–especially painkillers and different psychopharmacological drugs with the association being most prevalent early in pregnancy. Considering what can and do go wrong during the various stages of labour and delivery and considering how few connections we found between the factors examined and the later global QOL, it seems that the child is remarkably resilient to external influences during pregnancy and delivery concerned with global QOL, as an adult.

## INTRODUCTION

The concept of quality of life has been subject to a number of philosophical and psychological studies[1-15] conducted at the Quality of Life Research Centre in Copenhagen over resent years. It has been a subject of public debate[16-18] and a number of publications have published results concerning the association between quality of life (QOL) and a number of different factors as part of a major investigation of more than 11,000 Danes[19-25]. As one of the findings, we found that illness was closely related to the subjective perception of QOL, and therefore, the exploration of indicators related to QOL appears to be of significant importance for the prevention and treatment of diseases.

A series of potential dangers are known to threaten the child during pregnancy, delivery and birth. The hypothesis for the present study was that some of these risks have a negative influence on the quality of life of the adult child. This hypothesis was the reason for our investigation that analysed factors during pregnancy, delivery and birth in order to look for an association with the quality of life of the child 31-33 years later. The data for this study was from the Copenhagen Perinatal Birth Cohort 1959-1961[26], a cohort that has been the subject of many other prospective studies investigating the effects of pregnancy and birth on the child[27-61]. We used information from pregnancy, delivery and birth as indicators for describing links with the later self-reported quality of life of the child which to our knowledge has not been done before in any other studies.

## METHODS

The study is an analysis of the connection between different factors occurring during pregnancy, delivery and birth compared with the global quality of life (QOL) of the child 31-33 years later. These connections were based on two sets of responses. The first set was from the questionnaire filled out by the physician during pregnancy, delivery and birth and the second set was the grown-up child's own answers to the SEQOL questionnaire (Self evaluation of Quality of life Questionnaire)[62] at follow-up 31-33 years later.

The Copenhagen Perinatal Birth Cohort 1959-1961 began with the examination of 9,006 mothers and their 8,820 surviving children (from a total of 9,125 children) born at the State University Hospital in Copenhagen during the period 1959-1961[26-61]. With the help of the CPR-register (Central Person Register) we succeeded in tracing 7,222 of the original children from the cohort. To ensure that the people drawn from the register were actually the same persons in the original cohort, names and dates of birth were checked, as were the names and dates of birth of their mothers. When we repeatedly compare these data we found a match between the persons of around 98-99%, and therefore, estimate that the accurateness of the match of "the responses 1959-1961" against "the responses 1993" was better than 98%.

The SEQOL questionnaire is a self-administered questionnaire that measures the global quality of life with items rated on a five-point Likert scale. The questionnaire consists of 317 items and is based on an "integrative" theory of the quality of life. The integrative QOL theory was created to organize a number of theories on the quality of life into a spectrum that spans the extremes of subjective and objective quality of life. These measures are showed below (sample questions from the questionnaire included):

**Subjective measures**

1. Immediate, self-experienced well-being ("How are you feeling?")
2. Life satisfaction ("How satisfying is your life?")
3. Happiness ("How happy are you at present?")

**Existential measures**

4. Fulfilment of needs (e.g., "How well are your social needs fulfilled?")
5. Experience of life's temporal domains (e.g., "How do you feel when you are at home?")
6. Experience of life's spatial domains ("How satisfied are you with [each of five domains: self, partner, family, friends, community]"?)

7. Expression of life's potential [some 30 questions on extent to which they are fulfilled]

**Objective measure**

8.Objective factors [some 80 questions on income, status, work etc.]

Replies to each of the questions that constituted these measures were weighted and scored to yield computable numbers between a minimum of 0 and a maximum of 100[63-71]. These numbers were then taken as representing the quality of life of the respondent expressed in terms of the eight different ways the quality of life had been measured by the questionnaire. Suitably weighted and scored replies to the first part of the questionnaire constitute variables the co-variation of which the quality of life can be calculated.

Measuring quality of life has been the subject for many disagreements through time. In our research, the global QOL - in the most broad and all-including sense - is the primary outcome measure (dependent variable). The integrative QOL theory made us include 113 items in the SEQOL questionnaire for the calculation of the global QOL[72]. The rest were control questions and questions giving more information about health status, sexuality, philosophy of life, life style, self-perception and social circumstances. The principle of SEQOL is that of a hierarchy of factors adding up to an abstract total QOL.

In this study, we had to deal with an essential problem: When the statistical connection between 113 life factors and the global QOL was measured, we often had a contribution to the statistical co-variation from the construction of the global QOL measure. This problem turned out to be of little significance, as even the most strongly "constructed" connections did not count for more that $1/15^{th}$ of the total connection. Still, this gave an error of up to 7% in co-variation. As the large connections in our study showed a co-variation of 20% global QOL, or more, the above-mentioned error introduced by the construction of the global all-including QOL measure was generallynegligible. It is important to notice that the way our QOL measure was constructed it does not constitute a measuring problem, since we will almost always find a high correlation when N=5-10,000 between QOL and the many factors constituting the global QOL, or the factors related to them. However, we are not looking at the size of the correlation (the statistical significance), but at the size of the statistical co-variation (QOL difference in %) showing the clinical significance.

For validation, SEQOL was sent to 2,460 persons 18-88 year olds randomly selected from the Danish Central Person Register together with the Nottingham Health profile (NHP) and the Sickness Impact Factor (SIP). The test-retest reliability correlation was > 0.8, Cronbach's alpha was 0.75, correlation (r) to NHP was 0.49, to SIP 0.27 ($P<0.05$). Adjustment for health status made the correlation to SIP stronger among the sick ($r=0.41$). For SEQOL 111 respondents were needed to detect 3% difference in QOL. SEQOL are thus valid as it shows a high level of reliability, sensitivity and consistency.

## RESULTS

### Pregnancy

No significant associations were found between the quality of life of the adult child and the fact that the mother has been working with chemicals during pregnancy. Being born prematurely (before the 28th week of pregnancy), or post-maturely, did not show any connections either. No associations were found according to medical treatment received by the mother during pregnancy or to viral infections, which are often feared to cause possible harm to the foetus. However, the smoking habits of the mother were associated with the later quality of life. Children of mothers who smoked more than 10 cigarettes a day during the last trimester of pregnancy had a significantly lower quality of life as adults than children of mothers who were non-smokers (2.7% lower). Analysing the mother's medication during pregnancy showed that painkillers - especially early in pregnancy – were connected with a lower quality of life of the child as an adult (15.3% lower in the group where the mother took painkillers on at least 5 different occasions during the first month of pregnancy, see Table 1). Furthermore, the use of psychopharmacological drugs showed significant association with quality of life, the connection being smaller when the drugs were taken in the later stages of pregnancy. The mother's exposure to X-rays during pregnancy, which was not an uncommon occurrence before 1960, showed no correlation with the child's quality of life in later life and neither did weight gain, or oedema, in the mother during pregnancy, or the condition of the placenta, or the thickness of the subcutaneous fat layer of the newborn child. Apparently children were resilient to many of the complications occurring during pregnancy.

**TABLE I.**
**Pregnancy, delivery and birth**

The connection between global QOL and the mother's situation during pregnancy. Only statistically ($p<0.05$) and clinically significant factors listed. Difference in global QOL was measured according to the Integrated QOL theory[67] and measured with the validated SEQOL questionnaire[63].

| Quality of life and ... | QOL difference % * |
|---|---|
| ….mother's medication during pregnancy (painkillers in first month) | 15.3 |
| ...failed contraception (condom) | 3.8 |
| ... manner of delivery: expression (assistance during the birth) | 3.8 |
| ... mother with a mental illness during pregnancy (psychopharmacological medicine late in pregnancy) | 3.4 |
| ... Valgus position, one foot (versus nothing or both) | 3.2 |
| ... mother smoking during pregnancy (more than 10 cigarettes a day) | 2.7 |

* Difference in percentage between the worst and the best off (single events), or calculated with the method of weight modified linear regression (impact of all events)[71].

## First stage of birth (before expulsive stage)

In general, we found no connections between events in this stage and the quality of life of the child as an adult. Whether the birth was induced with medicine, whether it took less than 12 hours or more than 72 hours, whether there was a weak labour, whether there was mechanical hindrance, whether the mother was medicated, or anaesthetised, all appeared to be irrelevant for the quality of life of the child as an adult. The child seems to be very robust at this time of life.

## The expulsive stage

No associations were found between quality of life and complications during birth such as ruptured uterus, contracted pelvis in the mother, complications with the umbilical cord, pregnancy toxaemia, affected cardiac sound, discoloured amniotic fluid signifying a lack of oxygen during the birth, or whether the child was the first, or second, born twin (or triplet). The method of delivery also showed no connection with the quality of life in the adult. Only when the birth was aided by expression (outward pressure applied on the child through the mother's stomach) we found a connection with the quality of life (3.8% below the average). The foetal posture was not correlated with the quality of life of the child as an adult.

## DISCUSSION

Two main factors in pregnancy seemed to be associated with a reduced quality of life (QOL) for the child 31-33 years later: the mother's smoking habits and the mother's medication – especially painkillers and psychopharmacological drugs. From the point of view of prevention, it is interesting to note that in both cases the mother was in a position to control these factors. She could do without cigarettes during pregnancy and to a certain extent also without painkillers and psychopharmacological drugs.

Considering all the things which can and do go wrong during the various stages of labour, delivery and birth, and considering that no connection exists between these factors and QOL in later life, it seems that the child is remarkably resilient to external influences during birth, at least concerning later QOL. If we compare the potential dangers the child faces during pregnancy with the dangers faced during delivery and birth, the research implies that it is not the birth that is the real threat. Apparently, it is the pregnancy and especially factors such as smoking and the use of medicine that can diminish the quality of life of the child as an adult.

It is likely that in many cases it is not the medicine that is responsible for the later deterioration in QOL. Instead, the association can be described in terms of problems which necessitated the use of the medicine; for example, a low existential pain threshold as an indicator of a low quality of life[72,73] is strongly connected to a negative philosophy of life[74,75]. It is possible that this factor relats to the fact that consciousness[25] can be inherited in the form of non-constructive attitudes to life.

In previous studies, examining associations between global quality of life and close to 10,000 different indicators, we have shown that what is important for the global quality of life and health is good relations with the close as well as the distant world and the overall view of life of the person[17-23,25,74,75]. What one possesses in objective terms - money, status, work etc. - does not seem to be important for one's global quality of life and seems to be of little importance for self-assessed health. These results indicate that what is really important is not *what* you have, but *how you see, evaluate and experience what you have.* The person's level of consciousness and responsible attitude towards life and others seems far more important for the global quality of life and health than the person's social status and material wealth. According to these findings, it is not so strange that we only found a few strong associations between the numerous objective factors during the mother's pregnancy, delivery and birth and the global QOL of the adult child. The results seem to indicate that our lives as adults, only to a very small degree, are determined by the way we were exposed as a foetus and during birth, indicating that quality of life as adults is determined by what we ourselves choose to do with the capacities that we encompass. Our quality of life seems only to a marginal degree determined by our background and the capability given to us in the natural lottery, but is rather more determined by our attitude towards what happens to us.

An important limitation of the research is the quality of data. The 1959-1961 data was possibly sensitive to the opinion of the physician, and it is not known how uniform the research and data collection methods were at that time, even though a single physician did all the data collection during pregnancy. However, because of the very objective and standard form of the first set of data, an approximate uniformity is to a certain extent guaranteed. On the other hand, very important limitations of the data collected exist, because of their very objectivistic nature. It would have been valuable to include some subjective life evaluations from the mothers of the respondents in order to reveal more about the way in which her perceptions had an influence on the quality of life of her adult child.

## CONCLUSIONS

Only two main factors in pregnancy seem to be associated with a reduced quality of life for the child 31-33 years later: the mother's smoking habits and the mother's medication – especially painkillers and psychopharmacological drugs. Considering all that can go wrong during the various stages of labour, delivery and birth, and considering how very few connections existed between these factors and quality of life in later life, it seems that the child is remarkably resilient to external influences during birth. From the point of view of prevention, it is interesting to note that the factors that showed to be associated with the global quality of life were factors that the mother was in a position to control by stopping smoking and reducing her intake of painkillers and psychopharmacological drugs. Besides these preclusions it seems as if the quality of life of the adult child cannot be improved by any other precautions. As previous studies have shown it seems that global quality of life as an adult is only in a very limited way determined by background and early circumstances, and instead, are determined by the way you evaluate your life.

## ACKNOWLEDGEMENTS

This study was supported by grants from The 1991 Pharmacy Foundation, as well as by supplementary grants from Goodwill-fonden, the JL-Foundation, E. Danielsen and Wife's Foundation, Emmerick Meyer's Trust, the Frimodt-Heineken Foundation, the Hede Nielsen Family Foundation, Petrus Andersens Fond, Wholesaler C.P. Frederiksens Study Trust, Else & Mogens Wedell-Wedellsborg's Foundation and IMK Almene Fond. The research was approved by the Copenhagen Scientific Ethical Committee under number (KF) V.100.2123/91.

## BIOSKETCHES

**Søren Ventegodt, MD,** is the director of the Nordic School of Holistic Health and Quality of Life Research Center in Copenhagen, Denmark. He is also responsible for a Clinical Research Clinic for Holistic Medicine in Copenhagen and used as a popular speaker throughout Scandinavia. He has published numerous scientific or popular articles and a number of books on holistic medicine, quality of life and quality of working life. His most important scientific contributions are the comprehensive SEQOL questionnaire, the very short QOL5 questionnaire, the integrated QOL theory, the holistic process theory, the life mission theory, and the ongoing Danish Quality of Life Research Survey, 1991-94 in cooperation with the University Hospital of Copenhagen and the late professor of pediatrics, Bengt Zachau-Christiansen, MD, PhD. E-Mail: ventegodt@livskvalitet.org. Website: www.livskvalitet.org

**Trine Flensborg-Madsen**, BSc, is a research assistant of the Quality of Life Research Center in Copenhagen. She is currently finishing her Master of Public Health (MPH) at the Department of Public Health, University of Copenhagen, Denmark. E-mail: tfm@livskvalitet.org

**Niels Jørgen Andersen,** MSc, professor, Department of Innovation and Economic Organization, Norwegian School of Management. This department conducts research and provides teaching in central topics related to innovation, business development, management of global companies, business history and economic organization. Research activities within the Department are related to four core subjects within the discipline: business history, co-operative organizations, business development and entrepreneurship, and finally studies of industries with a special focus on the electricity industry. He is also the dynamic chairman of the non-profit organisation Stiftelsen Holistisk Medisin Scandinavia, that aims to support the scientific development, research, and documentation of complementary and holistic medicine in Scandinavia. E-mail: niels.j.andersen@bi.no. Website: http://www.bi.no/users/fgl93013

**Joav Merrick,** MD, DMSc, is professor of child health and human development affiliated with the Center for Multidisciplinary Research in Aging, Zusman Child Development Center, Division of Pediatrics and Community Health at the Ben Gurion University, Beer-Sheva, Israel, the medical director of the Division for Mental Retardation, Ministry of Social Affairs, Jerusalem, the founder and director of the National Institute of Child Health and Human Development. Numerous publications in the field of child health and human development, rehabilitation, intellectual disability, disability, health, welfare, abuse, advocacy, quality of life and prevention. Received the Peter Sabroe Child Award for outstanding work on behalf of Danish Children in 1985 and the International LEGO-Prize ("The Children's Nobel Prize") for an extraordinary contribution towards improvement in child welfare and well-being in 1987. E-Mail: jmerrick@internet-zahav.net. Website: www.nichd-israel.com
